# Redefining disease emergence to improve prioritization and macro-ecological analyses

**DOI:** 10.1016/j.onehlt.2015.08.001

**Published:** 2015-08-11

**Authors:** Samantha R. Rosenthal, Richard S. Ostfeld, Stephen T. McGarvey, Mark N. Lurie, Katherine F. Smith

**Affiliations:** a Department of Epidemiology, School of Public Health, Brown University, 121 S Main Street Box G-S121-2, Providence, RI 02912, United States; b Cary Institute of Ecosystem Studies, Box AB, Millbrook, NY 12545, United States; c International Health Institute, School of Public Health, Brown University, Box G-121-S, Providence, RI 02912, United States; d Department of Ecology and Evolutionary Biology, Brown University, 80 Waterman St. Box G-W, Providence, RI 02912, United States

**Keywords:** Disease ecology, Emerging pathogen, Pandemic, Emerging infectious disease

## Abstract

Microbial infections are as old as the hosts they sicken, but interest in the emergence of pathogens and the diseases they cause has been accelerating rapidly. The term ‘emerging infectious disease’ was coined in the mid-1900s to describe changes in disease dynamics in the modern era. Both the term and the phenomena it is meant to characterize have evolved and diversified over time, leading to inconsistencies and confusion. Here, we review the evolution of the term ‘emerging infectious disease’ (EID) in the literature as applied to human hosts. We examine the pathways (e.g., speciation or strain differentiation in the causative agent vs. rapid geographic expansion of an existing pathogen) by which diseases emerge. We propose a new framework for disease and pathogen emergence to improve prioritization. And we illustrate how the operational definition of an EID affects conclusions concerning the pathways by which diseases emerge and the ecological and socioeconomic drivers that elicit emergence. As EIDs appear to be increasing globally, and resources for science level off or decline, the research community is pushed to prioritize its focus on the most threatening diseases, riskiest potential pathogens, and the places they occur. The working definition of emerging infectious diseases and pathogens plays a crucial role in prioritization, but we argue that the current definitions may be impeding these efforts. We propose a new framework for classifying pathogens and diseases as “emerging” that distinguishes EIDs from emerging pathogens and novel potential pathogens. We suggest prioritization of: 1) EIDs for adaptation and mitigation, 2) emerging pathogens for preventive measures, and 3) novel potential pathogens for intensive surveillance.

## ‘Emerging infectious disease’ — evolution of a term

“*I always like informally to define emerging infections as those that would knock a really important story off the front page of the newspaper.*”

Stephen Morse [1]

Infectious disease events in humans, such as the initial outbreaks of measles in early agrarian societies 11,000 years ago, the geographic scope of the Black Death in the 14th century, and the introduction of smallpox to the New World in the 1500s undoubtedly would have made the ‘front page’ news of the time. These events have shaped human history for millennia, yet a growing body of literature suggests that host–pathogen dynamics are changing and giving rise to a novel cohort of ‘emerging infectious diseases’ (EIDs). Conceptualizations and definitions of EIDs have evolved in recent decades, affecting how epidemiologists and others interpret the causes and consequences of disease emergence. Below we explore the changing definitions of EIDs and their consequences, and offer an alternative framework that we hope will stimulate new efforts to better prioritize proactive and reactive approaches to disease emergence.

The earliest publications with ‘emerging disease’, ‘emerging pathogen’, or variations thereof in the title appeared in the 1950s and focused primarily on single disease events in livestock. Among these is a 1962 report on the introduction of Equine Piroplasmosis into the United States [2]. The paper's title, *Equine Piroplasmosis — Another Emerging Disease*, suggests that emergence was already a recognized phenomenon by the early 1960s. The 1970s and 1980s saw reports on EIDs in humans, livestock, pets, and in association with food crops [3], [4], [5], [6], [7], [8]. The first review of the topic, published in 1971 [9], chronicled the important EIDs of the time (cholera, diphtheria, gonorrhea, cryptococcosis, malaria, and hemorrhagic fevers to name a few), but did not provide a specific definition for emerging infectious diseases or emerging pathogens. Nevertheless, the final sentences established what most EID researchers would agree with today — change is to be expected. “*The microbiological system is closely allied with man; changes in the environment alter his relationship with organisms whether they be beneficial, symbiotic, or pathogenic. Man's way of life, his human behavior, his technological advances, his mere existence foster the conquest of some disease organisms, the emergence of others, and his introduction to unfamiliar ones. The infectious disease picture, therefore, is as subject to change as life itself* [9]*.*”

**Fig. 1:**
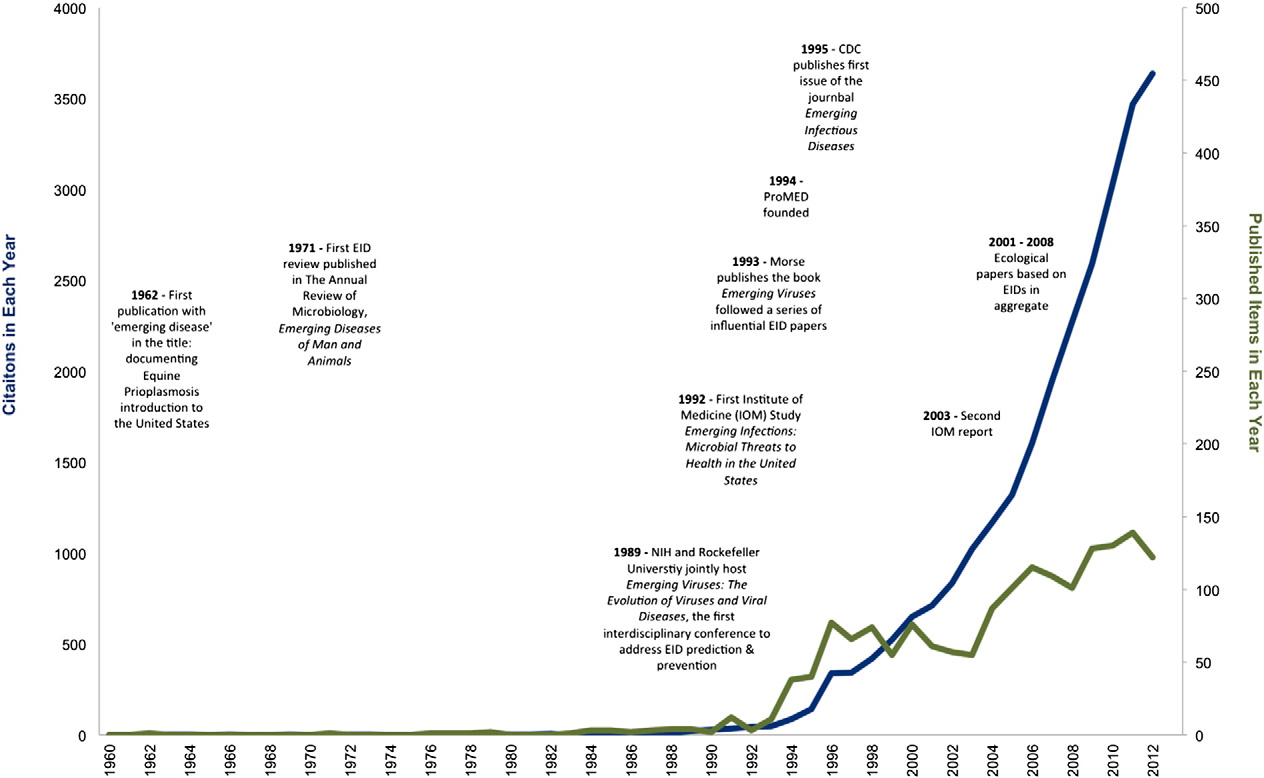
Emerging infectious disease publications and citations over time. We searched the Science Citation Index Expanded (ISI Web of Science) for papers published from 1900 to 2013 with English titles containing specific disease and pathogen emergence terms. Abstracts are not reliably available before 1990 so only titles were searched for 1900 to 1990. Our advanced search string was as follows: TI = (“emerging infect*”) OR TI = (“emerging disease*”) OR TI = (“emerging pathogen*”) OR TI = (“emerging virus”) OR TI = (“emerging bacteria”) OR TI = (“emerging helminth”) OR TI = (“emerging parasit*”) OR TI = (“emerging fung*”). Returned articles were used to create a graphic illustration of the number of published reports and citations of these reports in each year. Events, reports and publications influential in the development of the field if emerging infectious diseases are noted.

It was not until the late 1980s/early 1990s that organized scientific groups like the Institute of Medicine (IOM) became publically concerned with EIDs [10], [11]. Institutional interest in EIDs manifested through conferences, reports, and publications that sparked multi-disciplinary focus on the topic and set the stage for the surge in research that followed (Fig. 1) [10], [12], [13]. Two scientists in particular were especially influential in these years, Stephen Morse and Joshua Lederberg. Morse's work on viruses provided some of the first published definitions of emergence. “*We may use the term ‘emerging viruses’ to refer to viruses that either have newly appeared in the population or are rapidly expanding their range, with a corresponding increase in cases of disease* [12], [14].” Morse's opinion was that despite appearances, emerging viruses are often not newly evolved organisms, but instead are existing viruses in the process of invading new host groups or regions, a process he called ‘viral traffic’[12], [14].

Arguably, it was the 1992 IOM study, *Emerging Infections: Microbial Threats to Health in the United States*, co-authored by Lederberg, Robert Shope and Stanley Oaks [10], that launched the current phase of research on patterns, causes and consequences of emerging infectious diseases. The establishment of the Program for Monitoring of Emerging Diseases (ProMED) and the Centers for Disease Control and Prevention's (CDC) journal *Emerging Infectious Disease*s soon followed, as did initial research aimed at identifying the general characteristics and drivers of emerging diseases [15], [16]. EIDs were defined in the IOM (1992) report as “*clinically distinct conditions whose incidence in humans has increased*” while re-emergence was defined as “*the reappearance of a known disease after a decline in its incidence*” [10]. The CDC was the first to add a timeframe to the definition, promulgating EIDs as “*diseases of infectious origin whose incidence in humans has increased within the past two decades or threatens to increase in the near futu*re [11]”.

The first attempts to identify which of the more than 1400 infectious agents known to humans were causing, or were likely to cause, emerging diseases and to use these lists to identify EID risk factors occurred in the 21st century. Seminal work conducted during this period began to shed light on the myriad linkages between human, wildlife and livestock hosts that result in pathogen spillover (successful transmission from one host species to another) and subsequent emergence events [17]. Taylor et al. [18] produced the first list and quantitative study of emerging pathogens (not diseases) which they defined as, “… *those that have appeared in a human population for the first time, or have occurred previously but are increasing in incidence or expanding into areas where they had not previously been reported, usually over the last 20 years*.” They noted, “*Some definitions of emerging also include recently discovered aetiological agents of already-described diseases. However, if there was no evidence that such a pathogen was increasing in incidence, it was not regarded in this database as emerging.*” Their tally identified 175 pathogenic species associated with emerging diseases, 75% of which were zoonotic (transmitted to humans from non-human vertebrates). Viruses and protozoa were deemed especially likely to emerge while helminthes were not, and no effect of transmission type (i.e., vector-borne vs. direct transmission) was detected. This [18] research paved the way for additional studies on the risk factors for human disease emergence. One of these studies updated the list assembled by Taylor et al., examined the relationship between host range and pathogen emergence, and identified social and environmental causal factors, i.e., “drivers” [19]. This study also revealed that emerging zoonotic pathogens can be maintained in a wide variety of non-human hosts, tend to have broad host ranges, and are largely the result of human impacts on the landscape (e.g., deforestation to support agriculture and livestock) and changing human demographics (e.g., population density in particular) [19].

The definition of emerging infectious disease continued to evolve with the 2003 update of the 1992 IOM report: “*… either a newly recognized, clinically distinct infectious disease, or a known infectious disease whose reported incidence is increasing in a given place or among a specific population*” [20]. Five years later Jones et al. [21] published what is among the most cited EID papers to date. The authors presented the first spatiotemporal analysis of EID events (the original case or cluster of cases representing an infectious disease emerging in human populations for the first time) and their causal factors. They defined an emerging infectious disease as that which has “*recently increased in incidence, impact or geographic range. Specifically, it is caused by a pathogen that has recently evolved or entered the human population for the first time, or which has occurred previously, but is increasing in incidence or expanding into an area in which it has not previously been reported, or which has significantly changed its pathological or clinical presentation.*” Their compilation and analyses of 335 EID events was novel in a few ways. First, the report provided quantitative evidence for the long-held assumption that EIDs were increasing over time in the human population (since 1940). Second, each individual drug-resistant microbial strain was counted as a unique pathogen in the dataset, leading to the new finding that bacteria were more likely to be emerging pathogens than viruses. Third, using a method that accounted for an expected geographically-based bias in the reporting of EIDs, potential future EID hotspots were predicted in low and middle income countries where high human population density and biological diversity overlap; the very regions of the world where disease surveillance is most lacking [21].

Advances in infectious disease research have been rapid and multi-disciplinary in the last twenty years, and have been influential in resource allocation to improve EID prediction and prevention (e.g., USAID's Emerging Pandemic Threats Program [22]). What are the next steps for the field? How can the infectious disease research community better understand the macro-ecology of emerging infectious diseases? How can we sharpen our focus to be better at preventing and responding to the next influenza strain, coronavirus or Ebola outbreak? The working definition of emerging infectious diseases and pathogens plays a crucial role in efforts to do so. An evolving and vague or inconsistent definition is likely impeding these efforts [23].

In the sections below, we: 1) examine the pathways (how) by which diseases emerge; 2) propose a new framework for emergence to inform prioritization efforts; and 3) illustrate how the operational definition of an EID affects conclusions concerning the ecological and socioeconomic drivers (e.g., land use change, antimicrobial agent use) that elicit emergence.

## Pathways to emergence

The attributes that constitute emergence among pathogens and diseases have varied over time and space, as well as between studies. The medical and public health fields tend to focus on emerging diseases [10], [20], while ecologists are largely interested in their causal agents — emerging pathogens [18], [19], [21]. By chronicling changes in the definition of EIDs in the literature, we identified seven ‘pathways’ that researchers have used to identify diseases and pathogens as emerging in humans: 1) when a disease increases in incidence, 2) when a disease increases in impact (as measured, for example, by the associated disability in terms of morbidity and mortality), 3) when a disease increases in geographic range, 4) when a pathogen has undergone recent evolutionary change, 5) when a pathogen is detected the human population for the first time, 6) when a pathogen significantly changes its pathology or clinical presentation, or 7) when a pathogen is discovered for the first time. Collectively, these pathways capture nearly all the ways in which pathogens and the diseases they cause can change in the human population. These seven pathways have been used predominantly to identify emergence, but have been overlooked in macro-scale analyses of EIDs [18], [19], [21]. Neglect of the pathways used to designate diseases as emerging has left some important questions unanswered. Do most EIDs emerge via one or multiple pathways? Are some pathways more common than others in the emergence process? We consider these questions using a subset of chronicled EID events.

We took a disproportionate random sample from the database of 335 pathogens reported by Jones et al. [21] as causing EID events since 1940. Using Stata 10.0, we selected a sample with equal numbers of viruses, both zoonotic (n = 20) and non-zoonotic (n = 20), and bacteria, both zoonotic (n = 20) and non-zoonotic (n = 20), yielding a total of 80 events. A disproportionate sample was selected in this manner to increase the power to detect associations between pathogen type or host type and pathways or drivers. We focused on bacteria and viruses as these taxonomic groups are best studied (e.g., as compared to emerging helminths) and represent the majority of EID events.

Literature surveys were conducted to identify the pathways associated with each of the 80 EID events. The original references cited in Jones et al. were reviewed first, followed by PubMed and Google Scholar searches for the specific event until sufficient information was compiled to categorize events by one, some, or all of the seven proposed pathways of emergence. After examining references from Jones et al., if the disease pathogen could not be categorized sufficiently, we then searched PubMed by the name of the pathogen, followed by the year of the event, noted in Jones et al. If the pathogen still could not be sufficiently categorized based on results from the PubMed search, the same searches were conducted using Google Scholar. For a more detailed explanation of EID events, associated references, and assigned pathways see the supplemental information.

Our findings suggest that emergence rarely occurs as a result of only one pathway (Table 1). In fact, all of the 80 events we examined emerged via two to three pathways (38 events by two pathways, 33 events by three pathways, 9 events by four pathways). More than 40% of events were characterized by the pathways ‘detected in the human population for the first time’ and ‘newly discovered’. The only pathway not associated with any EID event in our sample was ‘changing in pathology or clinical presentation’. The broad way in which EIDs have been identified previously (by demonstrating one or more pathways of emergence) has the potential to mask critically important heterogeneity in these events, their drivers, and future hotspots of EID events.

**Table 1:** Disease emergence pathways.

Pathways of emergence	EID events N (%)
1. Increasing in incidence	80 (100)
2. Increasing impact	31 (39)
3. Increasing in geographic range	7 (9)
4. Newly evolved	23 (29)
5. Detected in the human population for the first time	34 (43)
6. Changing pathology or clinical presentation	0 (0)
7. Newly discovered	36 (45)

## A new framework for classifying emergence

**Fig. 2:**
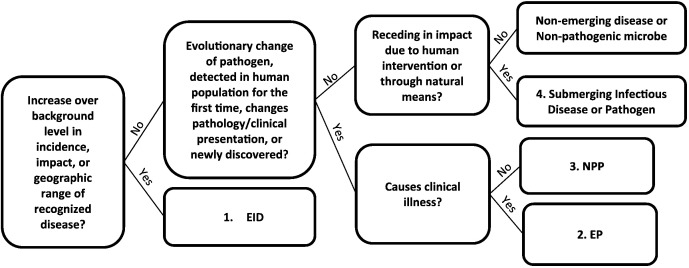
A decision tree applying a new framework for emergence. We redefined EID events as: 1. EIDs: those that increase in impact or increase in geographic range. 2. Emerging pathogens (EPs): those that have undergone recent evolutionary change, are entering detected in the human population for the first time, a pathogen has significantly changed its pathology or clinical presentation, or are newly discovered and show evidence that their presence in a human host causes clinical illness. 3. Novel potential pathogens (NPPs): those that are characterized by recent evolutionary change, are entering detected in the human population for the first time, or are newly discovered, but and show no evidence that their presence in a human host causes clinical illness. 4. Submerging infectious disease or pathogen: those that are receding in impact due to human intervention or through natural means.

To open a dialog on how the definition of EID affects the interpretation of drivers and biogeography of emergence, we here propose a new definitional framework. Our framework distinguishes between four stages of emerging diseases and pathogens and ranks them in decreasing order of immediate public health impact for prioritization purposes: 1) emerging infectious diseases, 2) emerging pathogens, 3) novel potential pathogens, and 4) submerging infectious diseases and pathogens. We present the framework in a decision tree (Fig. 2) and apply it to the subset of EID events described in the preceding section.

### Emerging infectious diseases (EIDs)

Emerging infectious diseases (EIDs) are those that have a clear detrimental impact on a human host population. Impact can occur both over space and time and is an inherent outcome of the spread of infectious disease to new human hosts. The pathways that constitute an emerging infectious disease are the three described in the most recent literature: 1) when a disease increases in incidence, 2) when a disease increases in impact, and 3) when a disease increases in geographic range. The question raised above, what constitutes impact, is key here. The impact of any given infectious disease can vary greatly between host individuals, populations, and places. Highly virulent pathogens, those that are challenging to treat, and those with high basic reproductive numbers (R_0_: the average number of cases one infected individual causes in a completely susceptible population) can result in widespread morbidity and mortality that in the worst cases also cause public unrest and economic loss. SARS and Ebola serve as examples of EIDs. Moving quickly via the commercial aviation network, SARS spread to 29 countries and infected more than 8000 people in eight months, costing the global economy 30–100 billion USD [24]. SARS in 2002–2003 was ‘emerging’ due to its increase in geographic range, impact and incidence. Similarly, the emergence of Ebola virus in several parts of Africa is another clear case of an EID. The original outbreaks in the Democratic Republic of Congo (then Zaire) and South Sudan (then Sudan) in 1976 involved > 600 cases with > 70% case fatality. Periodic outbreaks in Central Africa continued throughout the late 20th and early 21st Centuries, affecting dozens to hundreds of people each time, with similarly high fatality rates [25]. The first cases of the current Ebola virus outbreak were confirmed in March 2014, and as of the end of October 2014 there were > 6500 cases in Liberia, > 5300 in Sierra Leone, and > 1600 in Guinea, with other laboratory-confirmed cases in Mali, Senegal, Nigeria, Spain, and the United States [26], [27]. Ebola clearly meets the definition of an EID given the increase in the disease's incidence, impact (in terms of morbidity and mortality) and geographic range.

### Emerging pathogens (EPs)

Emerging pathogens (EPs) are those characterized by the following pathways: 4) when a pathogen has undergone recent, rapid evolutionary change tantamount to speciation or strain differentiation, 5) when a pathogen is detected the human population for the first time, 6) when a pathogen significantly changes its pathology or clinical presentation, or 7) when a pathogen is discovered for the first time. Emerging pathogens are distinguished from the next stage, novel potential pathogens, in that there is clear evidence that they can cause disease in humans. Relative to emerging infectious diseases, however, emerging pathogens (in their present state) do not cause significant levels of morbidity or mortality. This is not to imply that they do not have the *potential* to become an emerging infectious disease; some will and some may never. In many cases emerging pathogens will have been identified as infectious in a single or small number of local cases. Examples of emerging pathogens include many multi-drug resistant microbes. For these pathogens, the potential health impact will vary with the availability and accessibility of treatment options. Another example of an emerging pathogen is Jamestown Canyon virus. Jamestown Canyon virus is a member of the California serogroup of bunyaviruses spread by mosquitoes from the typical cervid reservoir. Though this pathogen was originally discovered in the 1960s in mosquitoes, only 15 human cases have been reported in the United States since 2004 [28]. The Jamestown Canyon virus is rare, yet endemic in parts of the United States and Canada. Though it was newly discovered in the 1960s and now found to cause illness in humans, it is not increasing in incidence, impact, or geographic range. Therefore, Jamestown Canyon virus is an emerging pathogen that should be targeted for surveillance and prevention measures, but not for the wide-scale mitigation efforts an EID would require.

### Novel potential pathogens (NPPs)

Novel potential pathogens (NPPs) are also characterized by pathways 4, 5 and 7, but are distinct from emerging pathogens in that there is no evidence that their presence in a human host causes clinical illness. Novel potential pathogens might include opportunistic microbes that are typical members of a human host microbiome. Only under unusual circumstances might they become pathogenic; for example when the human host becomes immune-compromised, the microbiome is altered in a manner that allows a particular microbe to reach high abundances and become pathogenic [29], or virulence increases due to a genetic or physiological change. Other examples of novel potential pathogens are those newly discovered in humans, but which do not yet cause clinical illness. Simian foamy viruses (SFVs), retroviruses highly prevalent in several animal species, are one example. Studies in central Africa have demonstrated spillover of these viruses from primates to primate hunters though to date there is no evidence that infection causes clinical illness in the latter [30].

### Submerging infectious diseases and pathogens

Submerging infectious diseases and pathogens are those receding in scope and impact due to human intervention or through natural means. We have the power to reduce the impact, incidence and geographic scope of emerging infectious diseases over time and so submergence should be part of a prioritization framework. In a few extraordinary cases, infectious diseases of global concern have been eliminated from the planet. Smallpox was eradicated from the human population, Rinderpest was eradicated from ungulates, and the end of Guinea Worm looks to be within reach [31], [32], [33]. Not so long ago, Smallpox and Rinderpest were harmful emerging infectious diseases. Smallpox emerged in the New World during the Age of Exploration and went on to decimate Native American populations, whereas Rinderpest was introduced to Africa in the late 1800s and wiped out such large numbers of domestic and wild ungulates that the entire southern part of the continent experienced sweeping ecosystem change [34]. Both diseases submerged over time, became endemic, and thanks to tireless human effort are now a part of history. Submerging infectious diseases are overlooked by the current EID literature, but an important stage in this new framework is the power of science-based policy to reduce the impact, incidence and geographic scope of emerging infectious diseases.

## Applying the framework

We redefined the 80 EID events according to our new framework to illustrate how the definition of EIDs affects biological conclusions regarding both pathways and drivers of EIDs. When describing drivers we considered all drivers assigned to at least five EID events (in our subset of 80 EID events). This led to a categorical variable for drivers that included: industry changes, international travel & commerce, land use changes, antimicrobial agent use, human susceptibility to infection, and other. ‘Other’ was created by combining all other drivers that were assigned to fewer than five EID events (e.g., breakdown in public health measures, bushmeat consumption, climate & weather, human demographics & behavior, war & famine, and unspecified).

**Fig. 3:**
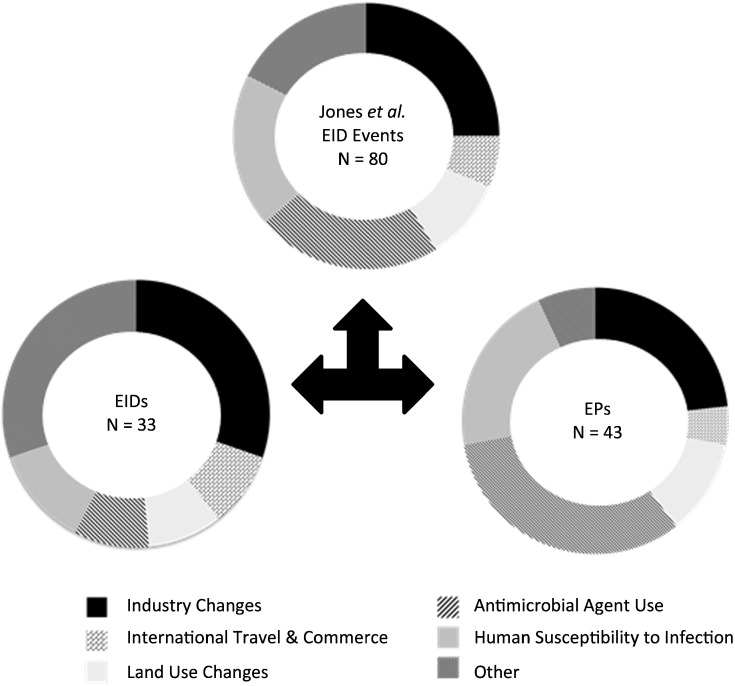
Difference in driver distribution among EIDs and EPs. Literature surveys of the 80 randomly selected EID events from Jones et al. [21] uncovered enough detail for us to apply the new framework (see supplementary information). This new classification revealed a significant difference in the distribution of causal drivers associated with EIDs versus EPs (p = 0.03).

After the reclassification of these EID events, only 41% of EID events considered maintained the classification of an EID under our framework. Over half (54%) were reclassified as EPs and 5% as NPPs (Table S1). Using Pearson's chi-square test, EIDs and EPs were shown to have significantly different drivers (p = 0.03; there were too few NPPs to consider; Fig. 3). Whereas EIDs were largely associated with industrial change, international trade and commerce, and ‘other drivers’, antimicrobial agent use was the dominant driver of EPs. This new classification demonstrates that the broad way in which EIDs have been categorized to date masks critically important heterogeneity in these events, their drivers, and therefore predictions of future occurrence (Fig. 3). These observations underscore the need for a consistent, transparent definition of EID and related concepts.

## Stage shifts in the emergence process

Our ability to identify a stage shift (e.g., when an NPP becomes an EP, when an EP becomes an EID, etc.) in the EID framework depends on three overarching conditions. First, detecting a shift depends on who is looking, when, and the factors that facilitate disease reporting, including scientific infrastructure, open media and internet usage [35], [36]. The shift of SARS from an emerging pathogen to an emerging disease took place over a matter of weeks. If Chinese public health officials had identified, contained and publically reported cases earlier, SARS might not have had the global impact that it did [37]. Recognizing that there will always be criticism during an epidemic, officials covering MERS-CoV seem to have benefitted from the lessons of SARS. Newly discovered cases are being reported in almost real-time to the public, and agencies (including the Saudi Arabian Ministry of Health, European Centre for Disease Prevention and Control, U.S. CDC and the WHO) are coordinating surveillance internationally.

Second, identifying a shift from emerging pathogen to emerging disease depends on how we choose to quantify significant increases and decreases in incidence, geographic range and impact. The research community is just now considering empirical methods to accomplish this. A recent proposal for segmented linear regression to examine changes in the incidence of a specific disease in a defined location is a promising start [38]. The next steps for such methods will be more effectively controlling for the influence of surveillance effort, discovery bias and reporting on incidence trends, adding a spatial dimension, and considering how best to calculate and track changes in impact. Related to this last point, the research community needs to decide which stages of emergence are most permeable to prevention and control efforts. This is the third condition that influences our ability to identify stage shifts.

Our literature review reinforces the notion that the distribution and impact of pathogens vary continuously in space and time, but when these facts should matter to public health is not straightforward. The economic costs and R_0_ values of EIDs are two metrics that have been used to quantify impact [39], [40], [41], [42]. R_0_ is typically calculated retrospectively, after diseases have emerged and are widely spread within a population and can vary geographically or between host populations. New methods from physics, based on the principles of network theory, offer the possibility of real-time estimates of R_0_ [43] and potential for assessing and comparing the impacts of emerging pathogens before they move to the next stage. While Disability Adjusted Life Years (DALYs) have been valuable in characterizing the global burden of many endemic and chronic infectious diseases, their application to EIDs remains notably absent but potentially promising in estimating impacts.

## Conclusion

We have reviewed the literature to describe the evolution of the term emerging infectious disease, identified the major pathways of emergence, presented a new framework for classifying pathogens and diseases at varying levels of emergence, and laid out the challenges associated with the current definition of EIDs. We have shown that the definition of an EID strongly affects our understanding of how and why diseases emerge, which in turn informs efforts to prevent and mitigate emergence. Ultimately, what the scientific and health communities want to understand is what causes pathogens and infectious diseases to become increasing threats so that they can either be prevented or mitigated quickly and efficiently. Having a consistent, operational, and transparent definition of emergence and related concepts is critical for this purpose, yet the current definition has drifted, becoming less operational and less consistent. The framework we propose suggests a prioritization scheme that focuses on pathogens with realized or likely imminent public health impact, but the impact threshold remains elusive. We suggest that EIDs should be prioritized for adaptation and mitigation, emerging pathogens for preventive measures, and novel potential pathogens for intensive surveillance. We think this new approach to emergence is likely to improve our ability to generate accurate generalizations about the patterns and processes governing trends in infectious disease, which will be critical for future efforts to protect global public health.

## Appendix A Supplementary data

The supplement contains the detailed methodology for assigning pathways to EID events, a list of EID events, associated references, and assigned pathways.
