# Supplementary material for: Redefining disease emergence to improve prioritization and macro-ecological analyses
Source: One Health. 2015 Aug 11;1:17–23. doi: 10.1016/j.onehlt.2015.08.001 (PMC5441331; doi:10.1016/j.onehlt.2015.08.001)
Supplement: Supplementary file 1 — The supplement contains the detailed methodology for assigning pathways to EID events, a list of EID events, associated references, and assigned pathways. [file mmc1.docx]

**SUPPLEMENTARY INFORMATION**

**Methodological details**

We assigned the pathway ‘increasing in incidence’ to any EID event with documentation of at least one new case of disease in the human population. In epidemiology, incidence is most broadly defined as the number of new cases per population over a specified time period. However, few publications report it that way, instead relying on the numerator (number of cases) as an index of disease magnitude or impact. Our decision to assign increasing in incidence to any event reporting at minimum one new case resulted in all events being characterized by this pathway. This is arguably an overestimate of significant disease rate increases, which could be corrected if pathogens are tracked through space and time–which is implausible to date given the lack of real-time surveillance data for any pathogen, let alone the whole human microbiome.

‘Increasing in impact’ was assigned when at least five new cases of disease were reported as occurring in the event. We chose number of cases as a proxy for impact since morbidity, mortality and economic effects were consistently not reported for the 80 EID events examined. Our criterion assumed that five new cases associated with a single emergence event resulted in substantial impacts to public health, although what justifies ‘substantial’ is highly subjective. Here again, our definition of impact was arbitrary because publications reported the cumulative number of cases inconsistently: studies indicated those having occurred in one nation, in multiple nations, or in a single hospital.

We categorized events as ‘increasing in geographic range’ if the pathogen was documented in a new nation or rediscovered in a previously endemic nation ≥10 years after its apparent absence. We focused on the national scale as it was the smallest geographic unit reported in all studies and the level at which public health infrastructures vary significantly. However, this definition fails to capture the reality that pathogens often appear in new regions within national borders. It also highlights the problem of determining ‘how far’ is far enough of a geographic expansion to call a pathogen emergent. Moreover, a pathogen that is increasing in geographic range should inherently also be increasing in incidence (and therefore also impact). This was effectively accounted for in our assignment given that all pathogens were determined to have emerged due to an increase in incidence but it serves as an example of redundancy in the current definition of an EID.

The pathway ‘recent evolutionary change’ includes pathogens that have become resistant to specific treatments and/or pathogens that have undergone mutations affecting pathogenicity. This definition was clearly identified in the literature documenting the 80 events examined. However, we would expect nations with greater surveillance capabilities to be more likely to detect and quickly document newly resistant strains, particularly those with large impacts, thus introducing a possible geographic bias. EID events ‘detected in the human population for the first time’ were also commonly identified. Many publications cite these pathogens as being the first clinical manifestations in humans. EID events with evolving pathogenesis or new strains/sub-types of known pathogens were not included in this definition for simplicity’s sake. Publication bias may have occurred when events that were not clearly stated to be the first clinical manifestation in humans were not included. ‘Newly discovered’ EID events included those pathogens that were detected in the human population for the first time, and those that were sub-types or new strains of disease. EID events were excluded from this mode if they had previously been isolated in animals or in humans without clinical presentation. Since the selected publication(s) did not all clearly indicate whether the pathogen had been previously isolated, misclassification could not be ruled out.

**Supplementary Table**

**Table S1.** EID events selected from Jones et al.^21^ that were reclassified following the new framework

| **Jones et al. Pathogen Name** | **EID Event Date** | **Place** | **Pathogen Type** | **Driver** | **Reclassification** | **Ref.** |
| --- | --- | --- | --- | --- | --- | --- |
| B19 Virus | 1979 | UK (London) | Virus | Other | EP | [44] |
| Barmah forest | 1985 | Australia (New South Wales) | Virus | International Travel & Commerce | EP | [45] |
| Bartonella elizabethae | 1986 | US (Brighton, Massachusetts) | Bacteria | Human Susceptibility to Infection | EP | [46] |
| Bordetella pertussis | 1993 | US | Bacteria | Industry Changes | EID | [47] |
| Brucella melitensis | 1995 | Malta and Gozo | Bacteria | Industry Changes | EID | [48] |
| Burkholderia pseudomallei | 1965 | Vietnam (Cu Chi) | Bacteria | Other | EID | [49] |
| California encephalitis | 1945 | US (California) | Virus | Land Use Changes | EP | [50] |
| Campylobacter jejuni fluoroquinolone-res | 1986 | Switzerland (Zurich) | Bacteria | Industry Changes | EP | [51] |
| Chlamydia trachomatis pandrug-res | 1997 | US (Atlanta, Georgia) | Bacteria | Antimicrobial Agent Use | EP | [52] |
| Crimean-Congo Hemorrhagic Fever | 1944 | Ukraine (Crimea) | Virus | Other | EP | [53] |
| Dengue | 1954 | Philippines (Manila) | Virus | Other | EID | [54] |
| Enterococcus faecium linezolid-res | 2000 | US (Chicago, Illinois) | Bacteria | Industry Changes | EID | [55] |
| Enterovirus 70 | 1969 | Ghana (Accra) | Virus | Other | EID | [56] |
| Escherichia coli non-O157:H7 | 1992 | Italy (Lombardia) | Bacteria | Industry Changes | EID | [57] |
| Escherichia coli O111:H- | 1951 | US (Virginia) | Bacteria | Industry Changes | EID | [58] |
| Escherichia coli O118:H12 | 1984 | Peru | Bacteria | Industry Changes | EID | [59] |
| Escherichia coli O163:H19 | 1984 | UK (Southampton) | Bacteria | Industry Changes | EP | [60] |
| Escherichia coli O45:H2 | 1983 | US (Michigan) | Bacteria | Industry Changes | EP | [61] |
| Escherichia coli O5:H- | 1980 | UK | Bacteria | Industry Changes | EP | [62] |
| Escherichia coli O55:H7 | 1980 | UK | Bacteria | Industry Changes | EP | [62] |
| Haemophilus influenzae | 2000 | Italy | Bacteria | Human Susceptibility to Infection | EID | [63] |
| Haemophilus influenzae chlor/tc-res | 1975 | Netherlands | Bacteria | Antimicrobial Agent Use | NPP | [64] |
| Hepatitis B HBeAg-negative (variant with truncated protein products) | 1989 | Italy; Greece | Virus | Industry Changes | EP | [65] |
| Hepatitis B vaccine escape mutant | 1990 | Italy (Neopolitan) | Virus | Other | EID | [66] |
| Hepatitis C | 1975 | US (Bethesda, Maryland) | Virus | Human Susceptibility to Infection | EP | [67] |
| Hepatitis G | 1964 | US (Illinois) | Virus | Industry Changes | EP | [68] |
| HIV- 1 Zidovudine-res | 1986 | US (San Diego, California) | Virus | Antimicrobial Agent Use | EP | [69] |
| HIV-1 multi-dideoxynucleoside res | 1994 | US (West Point, Pennsylvania) | Virus | Antimicrobial Agent Use | EP | [70] |
| Human enterovirus B Echovirus type 13 | 2000 | Spain (Canary Islands) | Virus | International Travel & Commerce | EID | [71] |
| Human Herpesvirus 1 Idoxuridine-res | 1963 | US (Gainesville, Florida) | Virus | Antimicrobial Agent Use | EP | [72] |
| Human Herpesvirus 3 acyclovir-res | 1986 | US (Suffolk County, New York) | Virus | Antimicrobial Agent Use | EP | [73] |
| Human Herpesvirus 5/CMV foscarnet-res | 1991 | US (Wisconsin) | Virus | Antimicrobial Agent Use | EP | [74] |
| Human Herpesvirus 5/CMV ganciclovir-res | 1989 | US (Minnesota) | Virus | Antimicrobial Agent Use | EP | [75] |
| Human Herpesvirus 6 | 1986 | US (Philadelphia, Pennsylvania) | Virus | Human Susceptibility to Infection | EID | [76] |
| Human Herpesvirus 7 | 1989 | US (Maryland) | Virus | Human Susceptibility to Infection | NPP | [77] |
| Human Herpesvirus 8 | 1994 | US (New York) | Virus | Human Susceptibility to Infection | EP | [78] |
| Human immunodeficiency virus 1 dideoxycytidine-res | 1990 | US (New Jersey) | Virus | Antimicrobial Agent Use | EP | [79] |
| Human T-Lymphotropic virus 1 | 1974 | Japan (Kyushu) | Virus | Human Susceptibility to Infection | EP | [80] |
| Influenza A virus | 1997 | China (Hong Kong) | Virus | Industry Changes | EP | [81] |
| Jamestown Canyon virus | 1960 | US (Wisconsin) | Virus | Land Use Changes | EP | [82] |
| Klebsiella pneumoniae | 1981 | Taiwan (T'ai-pei) | Bacteria | Human Susceptibility to Infection | EP | [83] |
| Kunjin virus | 1974 | Australia (Mildura, Victoria) | Virus | Land Use Changes | EP | [84] |
| LaCrosse virus | 1960 | US (Wisconsin) | Virus | Land Use Changes | EP | [85] |
| Legionella pneumophila | 1976 | US (Philadelphia, Pennsylvania) | Bacteria | Industry Changes | EID | [86, 87] |
| Leptospira fainei | 1995 | Australia (Victoria) | Bacteria | Industry Changes | EP | [88] |
| Machupo virus | 1959 | Boliva (El Beni) | Virus | Industry Changes | EID | [89] |
| Mayaro virus | 1954 | Trinidad | Virus | Land Use Changes | EID | [90] |
| Measles virus | 1990 | US | Virus | Other | EID | [91] |
| Menangle virus | 1997 | Australia (Sydney) | Virus | Industry Changes | EP | [92] |
| Mycobacterium abscessus | 1950 | US (Saint Louis, Missouri) | Bacteria | Human Susceptibility to Infection | EP | [93, 94, 95] |
| Mycobacterium celatum | 1989 | US (California) | Bacteria | Human Susceptibility to Infection | EID | [96] |
| Mycobacterium genavense | 1989 | Switzerland (Geneva) | Bacteria | Human Susceptibility to Infection | EID | [97] |
| Mycobacterium kansasii | 1955 | US (Dallas County, Texas) | Bacteria | Human Susceptibility to Infection | EP | [98] |
| Mycobacterium shimoidei | 1975 | Japan | Bacteria | Human Susceptibility to Infection | EP | [99] |
| Mycoplasma genitalium | 1981 | UK (London) | Bacteria | Other | EID | [100] |
| Neisseria gonorrhoeae fluoroquinolones-res | 1991 | Australia (Sydney) | Bacteria | International Travel & Commerce | EP | [101] |
| Neisseria gonorrhoeae tetracycline-res | 1983 | US (Nashua, New Hampshire) | Bacteria | Antimicrobial Agent Use | EID | [102] |
| Neisseria meningitidis serogroup W-135 | 2000 | Saudia Arabia | Bacteria | International Travel & Commerce | EID | [103] |
| Neisseria weaveri | 1960 | US (Washington) | Bacteria | Other | NPP | [104] |
| Norwalk virus | 1968 | US (Norwalk, Ohio) | Virus | Industry Changes | EID | [105] |
| Omsk virus | 1947 | Russia (West Siberia) | Virus | International Travel & Commerce | EID | [106] |
| Orungo virus | 1976 | Nigeria; Central Africa Republic | Virus | Other | EP | [107] |
| Picobirnavirus | 1984 | Brazil (Bahia) | Virus | Human Susceptibility to Infection | NPP | [108] |
| Poliovirus type 2 | 2003 | India (Uttar Pradesh) | Virus | Other | EID | [109] |
| Rabies virus | 2001 | Costa Rica (Gamba) | Virus | Land Use Changes | EID | [110] |
| Salmonella enterica serovar typhi cipro-res | 1991 | Nepal | Bacteria | Antimicrobial Agent Use | EID | [111] |
| SARS Coronavirus | 2001 | China (Guangdong Shen) | Virus | Other | EID | [112] |
| Serratia marcescens fluoroquinolone-res | 1986 | Taiwan | Bacteria | Antimicrobial Agent Use | EID | [113] |
| Shigella dysentatiae tet-res | 1951 | Japan | Bacteria | Antimicrobial Agent Use | EP | [114] |
| Shigella dysenteriae multiple drug-res | 1955 | Hong Kong | Bacteria | Antimicrobial Agent Use | EP | [115] |
| Shigella dysenteriae nalidixic ac.-res | 1981 | Congo (Kivu) | Bacteria | Antimicrobial Agent Use | EP | [116] |
| Shigella dysenteriae sulfa-res | 1949 | Japan | Bacteria | Antimicrobial Agent Use | EP | [117] |
| Sin Nombre virus | 1978 | US (Idaho) | Virus | Land Use Changes | EP | [118] |
| Sindbis virus | 1951 | Uganda (Waiya Bay) | Virus | Other | EID | [119] |
| Staphylococcus aureus penicillin-res | 1941 | US (Massachusetts) | Bacteria | Antimicrobial Agent Use | EP | [120] |
| Staphylococcus lugdunensis | 1988 | France (Lyon) | Bacteria | Human Susceptibility to Infection | EP | [121] |
| Streptococcus iniae | 1995 | Canada (Toronto) | Bacteria | Industry Changes | EID | [122] |
| Streptococcus pneumoniae macrolide-res | 1976 | France | Bacteria | Antimicrobial Agent Use | EP | [123] |
| Yersinia pestis | 1970 | US (New Mexico) | Bacteria | Land Use Changes | EID | [124] |
| Zika Virus | 1977 | Indonesia (Tegalyoso, Klaten) | Virus | Other | EID | [125] |

***References for Table S4***

1. Shneerson JM, Mortimer PP, Vandervelde EM. Febrile illness due to a parvovirus. *Br Med J* 1980; **280**: 1580.
2. Boughton CR, Hawkes RA, Naim HM. Illness caused by a Barmah Forest-like virus in New South Wales. *Med J Aust* 1988; **148**: 146-147.
3. Daly JS, et al. Rochalimaea elizabethae sp. nov. isolated from a patient with endocarditis. *J Clin Microbiol* 1993; **31**: 872-881.
4. Bass JW, Wittler RR. Return of epidemic pertussis in the United States*. Pediatr Infect Dis J* 1994; **13**: 343-345.
5. Amato Gauci AJ. The return of brucellosis*. Maltese Medical Journal* 1995; **7**: 7-8.
6. Chodimella U, Hoppes WL, Whalen S, Ognibene AJ, Rutecki GW. Septicemia and suppuration in a Vietnam veteran. *Hosp Pract (Minneap)* 1997; **32:** 219-21.
7. Hammon WM, Reeves WC. California encephalitis virus, a newly described agent. 1. Evidence of natural infection in man and other animals. *Calif Med* 1952; **77**: 303-309.
8. Adler-Mosca H, Altwegg M. Fluoroquinolone resistance in Campylobacter jejuni and Campylobacter coli isolated from human faeces in Switzerland. *J Infect* 1991; **23**: 341-342.
9. Somani J, et al. Multiple drug-resistant Chlamydia trachomatis associated with clinical treatment failure. *J Infect Dis* 2000; **181**: 1421-1427.
10. Watts DM, Ksiazek TG, Linthicum KJ, Hoogstraal H (1988) in *The Arboviruses: Epidemiology and Ecology*, Monath TP, Ed. (CRC Press, Boca Raton).
11. Hammon WM, Rudnick A, Sather GE. Viruses associated with epidemic hemorrhagic fevers of the Philippines and Thailand. *Science* 1960; **131**: 1102-1103.
12. Gonzales RD, et al. Infections due to vancomycin-resistant Enterococcus faecium resistant to linezolid. *Lancet* 2001; 357: 1179.
13. Chatterjee S, Quarcoopome CO, Apenteng A. Unusual type of epidemic conjunctivitis in Ghana. *Br J Ophthalmol* 1970; **54**: 628-630.
14. Caprioli A, et al. Community-wide outbreak of hemolytic-uremic syndrome associated with non-O157 verocytotoxin-producing Escherichia coli. *J Infect Dis* 1994; **169**: 208-211.
15. McKay DG. Shwartman phenomenon in fatal infantile diarrhoea due to excherichia coli O-111, B4. *Lancet* 1954; **264**: 1199-1200.
16. Wieler LH, et al. Enterohemorrhagic Escherichia coli (EHEC) strains of serogroup O118 display three distinctive clonal groups of EHEC pathogens. *J Clin Microbiol* 2000; **38**: 2162-2169.
17. Scotland SM, Rowe B, Smith HR, Willshaw GA, Gross RJ. Vero cytotoxin-producing strains of Escherichia coli from children with haemolytic uraemic syndrome and their detection by specific DNA probes. *J Med Microbiol* 1988; 25: 237-243.
18. Tzipori S, et al. Studies in gnotobiotic piglets on non-O157:H7 Escherichia coli serotypes isolated from patients with hemorrhagic colitis. *Gastroenterology* 1988; **94**: 590-597.
19. Dorn CR, et al. Properties of Vero cytotoxin-producing Escherichia coli of human and animal origin belonging to serotypes other than O157:H7. *Epidemiol Infect* 1989; **103**: 83-95.
20. Cerquetti M, et al. Invasive type e Haemophilus influenzae disease in Italy. *Emerg Infect Dis* 2003; **9**: 258-261.
21. van Klingeren B, van Embden JDA, Dessens-Kroon M. Plasmid-Mediated Chloramphenicol Resistance in Haemophilus influenzae. *Antimicrob Agents Chemother* 1977; **11**: 383-387.
22. Carman WF, et al. Mutation preventing formation of hepatitis B e antigen in patients with chronic hepatitis B infection. *Lancet* 1989; **2**: 588-591.
23. Carman WF, et al. Vaccine-induced escape mutant of hepatitis B virus. *Lancet* 1990; **336**: 325-329.
24. Feinstone SM, Kapikian AZ, Purcell RH, Alter HJ, Holland PV. Transfusion-associated hepatitis not due to viral hepatitis type A or B. *N Engl J Med* 1975; **292**: 767-770.
25. Simons JN, et al. Isolation of novel virus-like sequences associated with human hepatitis. *Nat Med* 1995; **1**: 564-569.
26. Larder BA, Darby G, Richman DD. HIV with reduced sensitivity to zidovudine (AZT) isolated during prolonged therapy. *Science* 1989; **243**: 1731-1734.
27. Shirasaka T, et al. Emergence of human immunodeficiency virus type 1 variants with resistance to multiple dideoxynucleosides in patients receiving therapy with dideoxynucleosides. *Proc Natl Acad Sci USA* 1995; **92**: 2398-2402.
28. Trallero G, et al. First epidemic of aseptic meningitis due to echovirus type 13 among Spanish children. *Epidemiol Infect* 2003; **130**: 251-256.
29. Field HJ. Herpes simplex virus antiviral drug resistance--current trends and future prospects. *J Clin Virol* 2001; **21**: 261-269.
30. Pahwa S, et al. Continuous varicella-zoster infection associated with acyclovir resistance in a child with AIDS. *JAMA* 1988; **260**: 2879-2882.
31. Knox KK, Drobyski WR, Carrigan DR. Cytomegalovirus isolate resistant to ganciclovir and foscarnet from a marrow transplant patient. *Lancet* 1991; **337**: 1292-1293.
32. Erice A, et al. Progressive disease due to ganciclovir-resistant cytomegalovirus in immunocompromised patients. *N Engl J Med* 1989; **320**: 289-293.
33. Salahuddin SZ, et al. Isolation of a new virus, HBLV, in patients with lymphoproliferative disorders. *Science* 1986; **234**: 596-601.
34. Frenkel N, et al. Isolation of a new herpesvirus from human CD4+ T cells. *Proc Natl Acad Sci USA* 1990; **87**: 748-752.
35. Chang Y, et al. Identification of herpesvirus-like DNA sequences in AIDS-associated Kaposi's sarcoma. *Science* 1994; **266**: 1865-1869.
36. Fitzgibbon JE, et al. Human immunodeficiency virus type 1 pol gene mutations which cause decreased susceptibility to 2',3'-dideoxycytidine. *Antimicrob Agents Chemother* 1992; **36**: 153-157.
37. Poiesz BJ, Ruscetti FW, Reitz MS, Kalyanaraman VS, Gallo RC. Isolation of a new type C retrovirus (HTLV) in primary uncultured cells of a patient with Sezary T-cell leukaemia. *Nature* 1981; **294**: 268-271.
38. Subbarao K, et al. Characterization of an avian influenza A (H5N1) virus isolated from a child with a fatal respiratory illness. *Science* 1998; **279**: 393-396.
39. Thompson WH, Evans AS. California Encephalitis Virus Studies in Wisconsin. *AJE* 1965; **81**: 230-244.
40. Cheng DL, Liu YC, Yen MY, Liu CY, Wang RS. Septic metastatic lesions of pyogenic liver abscess. Their association with Klebsiella pneumoniae bacteremia in diabetic patients. *Arch Intern Med* 1999; **151**: 1557-1559.
41. Doherty RL, Carley JG, Flippich C, White J, Gust ID. Murray Valley Encephalitis in Australia, 1974: Antibody Response in Cases and Community. *Aust N Z J Med* 1976; **6**: 446-453.
42. Thompson WH, Kalfayan B, Anslow RO. Isolation of California Encephalitis Group Virus from a Fatal Human Illness*. AJE* 1965; **81**: 245-253.
43. Ching WT, Meyer RD. Legionella infections. *Infect Dis Clin Noth Am* 1987; **1**: 595-614.
44. Steinert M, Heuner K, Hacker J (2001) in *Emerging Bacterial Pathogens,* Muhldorfer I, Schafer KP, Eds. (Karger, Basel) pp 12-19.
45. Chappel RJ, et al. Serological titres to Leptospira fainei serovar hurstbridge in human sera in Australia. *Epidemiol Infect* 1998; **121**: 473-475.
46. Mackenzie RB, Webb PA, Johnson KM. Detection of complement-fixing antibody after Bolivian Hemorrhagic fever, employing Machupo, Junin and Tacaribe virus antigens. *Am J Trop Med Hyg* 1965; **14**: 1079-1085.
47. Anderson CR, Downs WG, Wattley GH, Ahin NW, Reese AA. Mayaro virus: a new human disease agent. II. Isolation from blood of patients in Trinidad, B. W. I. *Am J Trop Med Hyg* 1957; **6**: 1012-1016.
48. Lederberg J, Shope RE, Oaks Jr SC, Eds. (1992) *Emerging infections: microbial threats to health in the United States* (National Academy Press, Washington, D.C.) pp 312.
49. Chant K, et al. Propbable human infection with a newly described virus in the family Paramyxoviridae. *Emerg Infect Dis* 1998; **4**: 273-273.
50. Buhler VB, Pollak A. Human infection with atypical acid-fast organisms; report of two cases with pathologic findings. *Am J Clin Pathol* 1953; **23**: 363-74.
51. Runyon EH. Anonymous mycobacteria in pulmonary disease. *Med Clin North Am* 1959; **43**: 273-290.
52. van Ingen J (2009), thesis, Radboud University.
53. Butler WR, et al. Mycobacterium celatum sp. nov. *Int J Syst Bacteriol* 1993; **43**: 539-548.
54. Bottger EC, et al. Disseminated "Mycobacterium genavense" infection in patients with AIDS. *Lancet* 1992; **340**: 76-80.
55. Christianson LC, Dewlett HJ. Pulmonary disease in adults associated with unclassified mycobacteria. *Am J Med* 1960; **29**: 980-991.
56. Tsukamura M, Shimoide H, Shaefer WB. A possible new pathogen of group iii Mycobacteria. *J Gen Microbiol* 1975; **88**: 377-380.
57. Thomassen MJ, et al. Pseudomonas cepacia colonization among patients with cystic fibrosis. A new opportunist. *Am J Respir Crit Care Med* 1985; **131**: 791-796.
58. Tapsall JW, et al. Failure of 500 mg ciprofloxacin therapy in male urethral gonorrhoea. *Med J Aust* 1992; **156**: 143.
59. Tetracycline-resistant Neisseria gonorrhoeae--Georgia, Pennsylvania, New Hampshire. *Morb Mortal Wkly Rep* 1985; **34**: 563-4, 569-70.
60. Lingappa JR, et al. Serogroup W-135 meningococcal disease during the Hajj, 2000. *Emerg Infect Dis* 2003; **9**: 665-71.
61. Andersen BM, et al. Neisseria weaveri sp. nov., formerly CDC group M-5, a gram-negative bacterium associated with dog bite wounds. *J Clin Microbiol* 1993; **31**: 2456-2466.
62. Adler JL, Zickl R. Winter vomiting disease. *J Infect Dis* 1969; **119**: 668-673.
63. Burke DS, Monath TP (2001) in *Field’s Virology, fourth ed.,* Knipe DM, Howley PM, Eds. (Lippincott Williams & Wilkins, Philadelphia) pp 1043–1125.
64. Tomori O, Fabiyi A, Murphy F. Characterization of Orungo Virus, an Orbivirus from Uganda and Nigeria*. Arch Virol* 1976; **51**: 285-298.
65. Ludert JE, Abdul-Latiff L, Liprandi A, Liprandi F. Identification of picobirnavirus, viruses with bisegmented double stranded RNA, in rabbit faeces. *Res Vet Sci* 1995; **59**: 222-225.
66. Grassly NC, et al. Asymptomatic wild-type poliovirus infection in India among children with previous oral polio vaccine. *J Infect Dis* 2010; **201**: 1535-1543.
67. Badilla X, et al. Human rabies: a reemerging disease in Costa Rica? *Emerg Infect Dis* 2003; **9**: 721-723.
68. Rowe B, Ward LR, Threlfall EJ. Ciprofloxacin-resistant Salmonella typhi in the UK. *Lancet* 1995; **346**: 1302.
69. Xu RH, et al. Epidemiologic clues to SARS origin in China. *Emerg Infect Dis* 2004; **10**: 1030-1037.
70. Sheng WH, et al. Emerging fluoroquinolone-resistance for common clinically important gram-negative bacteria in Taiwan. *Diagn Microbiol Infect Dis* 2002; **43**: 141-147.
71. Mitsuhashi S, Hashimoto H, Egawa R, Tanaka T, Nagai Y. Drug Resistance of Enteric Bacteria: IX. Distribution of R Factors in Gram-negative Bacteria from Clinical sources. *J Bacteriol* 1967; **93**: 1242-1245.
72. Feingold DS. Infectious drug resistance in enteric bacteria. Seminars in Medicine of the Beth Israel Hospital, Boston. *N Eng J Med* 1966; **275**: 888-894.
73. Malengreau M. Nalidixic acid in Shigella dysenteriae outbreaks. *Lancet* 1984; **324**: 172.
74. Kondo E, Mitsuhashi S. Drug resistance of enteric bacteria IV. Active Transducing bacteriophage P1 CM produced by the combination of R factor with bacteriophage P1. *J Bacteriol* 1964; **88**: 1266-1276.
75. Zaki SR, et al. Retrospective diagnosis of hantavirus pulmonary syndrome, 1978-1993: implications for emerging infectious diseases. *Arch Pathol Lab Med* 1996; **120**: 134-139.
76. Taylor RM, et al. Sindbis virus: a newly recognized arthropodtransmitted virus. *Am J Trop Med Hyg* 1955; **4**: 844-862.
77. Kirby WMM. Extraction of a highly potent penicillin inactivator from penicillin resistant staphylococci. *Science* 1944; **99**: 452-453.
78. Frener J, et al. Staphylococcus lugdunensis sp. nov. and Staphylococcus schleiferi sp. nov., two species from human clinical specimens. *Int J Syst Evol Microbiol* 1998; **38**: 168-172.
79. Weinstein MR, et al. Invasive infections due to a fish pathogen, Streptococcus iniae. S. iniae Study Group. *N Engl J Med* 1997; **337**: 589-594.
80. Geslin P, Buu-Hoi A, Fremaux A, Acar JF. Antimicrobial resistance in Streptococcus pneumoniae: an epidemiological survey in France, 1970-1990. *Clin Infect Dis* 1992; **15**: 95-98.
81. Hull HF, Montes JM, Mann JM. Septicemic Plague in New Mexico. *J Infect Dis* 1987; **155**: 113-118.
82. Olson JG, Ksiazek TG, Suhandiman, Triwibowo. Zika virus, a cause of fever in Central Java, Indonesia. *Trans R Soc Trop Med Hyg* 1981; **75**: 389-393.
